# Benefits and Challenges of Treat-to-Target in Inflammatory Bowel Disease

**DOI:** 10.3390/jcm12196292

**Published:** 2023-09-29

**Authors:** Jack West, Katrina Tan, Jalpa Devi, Finlay Macrae, Britt Christensen, Jonathan P. Segal

**Affiliations:** 1 Department of Gastroenterology, Royal Melbourne Hospital, Parkville, Melbourne 3052, Australia; 2 Department of Gastroenterology, Northern Health, Epping, Melbourne 3076, Australia; 3 Department of Gastroenterology, Washington University in Saint Louis, St. Louis, MI 63110, USA; 4 The University of Melbourne, Parkville, Melbourne 3010, Australia

**Keywords:** inflammatory bowel disease, treat to target, ulcerative colitis, crohn’s disease, endoscopy, mucosal healing

## Abstract

There is notable disparity between symptomatology and disease activity in a significant proportion of patients with inflammatory bowel disease (IBD), and escalation of treatment based on symptoms alone can fail to significantly alter the course of disease. The STRIDE-II position statement, published in 2021 by the Selecting Therapeutic Targets in Inflammatory Bowel Disease (STRIDE) initiative of the International Organisation for the Study of IBD (IOIBD) provides the most current recommendations for a treat-to-target (T2T) approach in IBD. Despite the benefits offered by a T2T approach in IBD, there are numerous drawbacks and current limitations to its widespread implementation in real-world clinical practice. Owing to the lack of a standardised definition of MH, outcome data are heterogeneous and limit the comparability of existing data. Further, studies investigating the likelihood of achieving MH with a T2T approach are limited and largely retrospective. Evidence of the real-world feasibility of tight monitoring is currently minimal and demonstrates sub-optimal adherence among patients. Further, the few studies on the acceptability and uptake of a T2T approach in real-world practice demonstrate the need for increased acceptability on both patients’ and clinicians’ behalf. Real-world applicability is further limited by the need for repeated endoscopic assessments of MH as well as a lack of guidance on how to incorporate the various treatment targets into therapeutic decision-making. We aim to review the benefits and challenges of the T2T approach and to discuss potential solutions to further patient care.

## 1. Introduction

Crohn’s disease (CD) and ulcerative colitis (UC) are chronic, immune-mediated diseases of the gastrointestinal tract. Encompassed under the term inflammatory bowel disease (IBD), these conditions carry significant morbidity with the potential for serious complications if not adequately treated [1,2]. Previous approaches to IBD treatment have largely focused on the resolution of symptoms alone, with a view to escalate the available therapies as needed to achieve steroid-free clinical remission. However, it is now recognised that a significant proportion of patients continue to have smouldering inflammation despite the resolution of symptoms and that treatment targeting the control of symptoms alone fails to significantly alter the natural history of these conditions [3].

The STRIDE-II position statement, an update on the original STRIDE guidelines published in 2015, provides treatment goals for a treat-to-target approach to managing IBD, derived from extensive review of the literature and expert consensus [4]. These guidelines support the establishment of patient-centred targets but are ultimately geared towards achieving the long-term composite goal of clinical and endoscopic remission. Numerous updates are provided in the STRIDE-II guidelines, including the stratification of targets into relevant time frames and the inclusion of normalised quality of life (QoL) and avoidance of disability as a long-term goal [4]. 

While studies have demonstrated improved patient outcomes with a treat-to-target approach in IBD, overall, the evidence is heterogeneous and predominantly retrospective [5]. Further, there remains uncertainty as to the real-world feasibility and widespread implementation of a treat-to-target approach beyond academic and IBD centres [6]. Herein, we offer a critical appraisal of a treat-to-target approach in IBD. We review the established benefits and numerous pitfalls of this approach and explore the challenges to its implementation in real-world clinical practice as well as some potential solutions. 

## 2. What Is Treat-to-Target and How Do We Achieve It?

**Figure 1:**
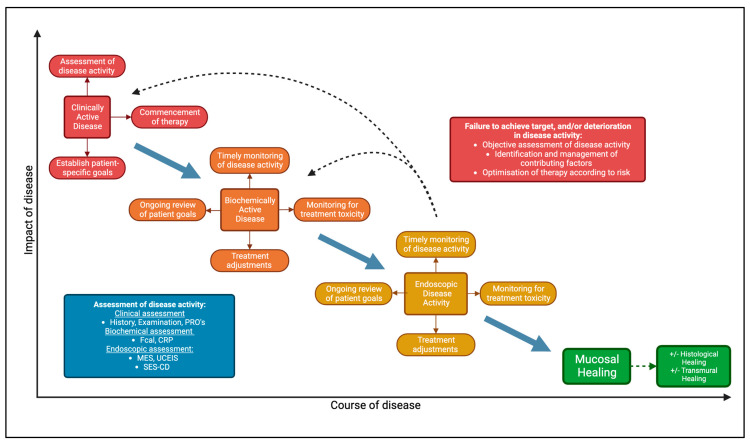
Overview of a treat-to-target approach in IBD. Patient-specific goals are defined and worked towards with tight monitoring of disease activity informing changes to treatment. Created with BioRender.com, accessed on 21 August 2023.

Treat-to-target (T2T) is a multifaceted concept that describes an approach to managing chronic disease. It has been successfully employed and demonstrated to improve patient outcomes in multiple chronic conditions such as rheumatoid arthritis, diabetes mellitus, and hypertension [7,8,9]. In the case of IBD, T2T involves directing treatment towards well-defined, patient-specific goals with a timely objective assessment of disease activity (“tight control”) informing tailored adjustments to treatment accordingly (Figure 1) [4]. Notwithstanding patient preference, the T2T approach is applicable to all patients with IBD, with the complexities and nuances in managing individual patients accounted for within the patient-specific design of the T2T concept. 

Formal treatment targets for the management of IBD were published in 2015 by the International Organisation for the Study of IBD (IOIBD) in the form of the STRIDE guidelines [10]. These guidelines were formed following an extensive literature review and consensus by an expert panel and were further updated in 2021 to form the STRIDE-II guidelines [4]. The guidelines encompass clinical, inflammatory biomarkers and endoscopic response as targets, stratified into relevant approximate timeframes. Whilst there are subtle differences in the treatment goals for CD and UC, the overarching treatment goals are similar. The long-term treatment targets recommended for both CD and UC are the composite endpoints of mucosal healing (MH) and clinical remission. Normalised QoL and the absence of disability are incorporated into the concept of clinical remission, reflecting the impact of IBD-associated disability on patients’ functional and psychosocial wellbeing. 

Prior to the concept of treating-to-target in IBD, treatment was predominantly geared towards achieving steroid-free control of symptoms, with the long-term view of preventing hospitalisation and colectomy [11]. It has subsequently been recognised, however, that deeper and more objective control of inflammation is needed in order to affect the course of disease and reduce structural damage and disability [6,10,11]. The STRIDE-II guidelines target long-term mucosal healing as an objective and specific reflection of disease control in both UC and CD. While transmural healing in CD and histological healing in UC are speculated to be potentially superior targets to mucosal healing, there is inadequate current evidence to support this, and as such, STRIDE-II incorporates these as informal, accessory targets [4].

Tight control involves regular and timely objective assessment of disease control [6]. Objective clinical assessment is aided by use of disease activity scores such as the Crohn’s disease activity index (CDAI) and the partial Mayo score (PMS) for UC [12]. Inflammatory biomarkers are employed as a surrogate for endoscopic disease activity, with C-reactive protein (CRP) and faecal calprotectin (Fcal) the most studied [6,13,14]. Normalisation of CRP and Fcal are incorporated into the STRIDE-II guidelines as intermediate targets. A failure of these biomarkers to normalise with directed treatment should thus prompt adjustments in therapy and/or more in-depth disease assessment as indicated in the clinical context [4]. Endoscopy remains the gold standard for disease assessment in IBD and is currently the only validated means of assessing MH, aside from capsule endoscopy in certain cases of CD [15]. The use of endoscopic scores such as the simple endoscopic score for Crohn’s disease (SES-CD) and the endoscopic Mayo score and the ulcerative colitis endoscopic index of severity (UCEIS) provides reproducibility and the quantification of mucosal disease [6]. With timely and objective assessment of disease activity post commencement of therapy, treatment can be adjusted accordingly, guiding disease control towards the pre-defined targets. 

## 3. Evidence Supporting Mucosal Healing

Despite heterogeneity in the definitions and assessment of MH, studies demonstrate that the achievement of the parameters of MH predicts better long-term outcomes for patients with UC and CD (Table 1). These outcomes include long-term clinical remission and steroid-free clinical remission, the avoidance of colectomy and other disease-related surgery, and the achievement of sustained, long-term MH. As such, mucosal healing is the predominant long-term treatment target for both CD and UC in the STRIDE-II guidelines [4]. 

## 4. Challenges of a Treat-to-Target Approach 

### 4.1. Need for Repeated Endoscopy 

The prognostic importance of MH and reliance on endoscopic assessment means that endoscopy remains vital to a successful treat-to-target approach. Three-monthly endoscopic assessment of mucosal healing should be undertaken during active disease for UC and assessment at 6–9 monthly intervals for CD according to the STRIDE guidelines [10]. For endoscopic assessment in clinical remission, the consensus recommends an interval based on screening recommendations in deep remission prompted by clinical symptoms or consistent Fcal positivity [6]. Multiple issues preclude repeated endoscopic assessments in real-world clinical practice. Endoscopic procedures are costly to healthcare systems and are limited in availability and accessibility, invariably involving waiting lists in public healthcare systems [23]. Endoscopy is also relatively invasive, and the benefits of repeated endoscopic assessment need to be weighed against the risks of bowel perforation and bleeding [24]. Further, the need for repeated sedating anaesthesia may preclude this approach in elderly patients as well as those with significant cardiorespiratory co-morbidities [25]. The current reliance on endoscopy for the assessment of MH limits the current feasibility of the widespread implementation of T2T approaches in real-world clinical practice. 

Adequate bowel preparation is essential for accurate endoscopic mucosal assessment in IBD, and the logistics and safety of this warrants consideration in a regimen of serial endoscopy [26]. A recent review of bowel preparation formulations in IBD populations found that polyethylene-glycol-based bowel preparation regimens appeared to have the best safety profile in IBD patients, with split regimens being preferred [27]. Caution is required in patients with active disease however, irrespective of the form of preparation due to the risk of mucosal damage with bowel preparation [27].

### 4.2. Lack of Universal and Validated Definition of Mucosal Healing 

There is a present lack of a standardised and validated definition of MH. The updated STRIDE-II guidelines recommend targeting MH as defined by SES-CD ≤ 2 points or the absence of ulcerations in CD, and MES = 0 or UCEIS ≤ 1 in UC [4]. These specific targets remain the result of expert consensus however, and they are yet to be fully validated in predicting specific disease outcomes [28,29]. 

The definitions of MH used in the existing literature are also heterogeneous, limiting the comparability of findings. In studies of UC, MH is commonly defined as MES ≤ 1; however, compared to MES 1, the achievement of MES 0 is associated with improved disease control. A recent meta-analysis of 15 studies of 1617 UC patients in steroid-free CR found patients with MES 0 had a pOR of 0.33 (95% CI, 0.26–0.43; I^2^ 13%; *p* < 0.00001) for clinical relapse irrespective of the time of follow-up compared to patients with MES 1 disease [30]. A similar meta-analysis of 17 studies and 2608 patients with UC in CR defined by a composite of PRO’s and MES scores of 0 or 1 found patients achieving MES 0 had a 52% lower risk of clinical relapse (relative risk, 0.48; 95% CI, 0.37–0.62) compared to patients with MES 1 [31]. 

The recommended endoscopic index thresholds in CD also remain empiric and unvalidated [32]. SES ≤ 2 as a definition of MH is the result of expert consensus from the IOIBD committee review on clinical trials [28]. The definitions employed in the literature remain varied however, and less significant improvements in MH have at times most strongly predicted clinical outcomes. For example, a post hoc analysis of the data from the SONIC trial demonstrated that endoscopic response at 26 weeks, defined as a decrease of ≥50% from baseline SES-CD, was more predictive of steroid-free clinical remission at 50 weeks than MH, defined as the complete absence of mucosal ulceration [19]. 

It remains unclear what degree of MH needs to be achieved in order to predict long-term clinically significant outcomes. Until a standardised and validated definition of MH exists for both UC and CD, the comparability of evidence and the widespread implementation of T2T strategies geared around endoscopic disease assessment will remain limited. Beyond endoscopic assessment, future research will be required to establish if the achievement of more advanced disease control parameters such as histological remission and transmural healing predict improved clinical outcomes. 

### 4.3. Evidence of Treat-to-Target Strategies Incorporating Serial Endoscopy Is Largely Retrospective 

The existing studies demonstrate that approximately 50% of IBD patients can achieve MH without a T2T strategy, with specific rates dependent on the intervention, definition of MH, and time of assessment [33]. There are currently no data comparing the rates of achieving MH between a T2T and symptom-based approach to IBD management. Further, the current evidence investigating the rate of achieving MH with a T2T strategy incorporating serial endoscopy is largely limited to retrospective cohort studies (Table 2). 

There are five published studies, four of which are retrospective cohort studies and one of which is a prospective observational study. Patient numbers range from 50 to 272 patients. In three of the studies, endoscopic findings were used to inform adjustments to treatment. The definition of MH used varied between all the studies, and MH was achieved in between 44.4% and 60% of patients across the studies at various time points. 

### 4.4. Feasibility of Tight Monitoring Utilising a T2T Strategy in Real-World Practice 

There are little data demonstrating the feasibility of close monitoring to inform treatment changes in a T2T approach in the real world. Further, the available evidence demonstrates a sub-optimal proportion of patients completing objective disease assessments at the designated intervals (Table 3). 

The data from the TARGET-IBD prospective longitudinal cohort study looked to assess the frequencies of objective disease assessments and therapeutic drug monitoring (TDM) being undertaken prior to changes in biologic therapies in IBD patients receiving usual care in America [40]. A total of 525 patients (71.4% CD, 28.6% UC) receiving a biologic therapy underwent either a dose change or discontinuation due to a lack of efficacy. A total of 292 patients underwent dose escalation, with 197 (67.5%) patients having at least 1 objective disease activity assessment in the 12 weeks prior, and 105 (36.0%) having ≥2. Rates did not differ between UC or CD. The most common means of disease assessment was CRP (39.1% in CD and 54.5% in UC), whereas Fcal was much less utilised (5.6% in CD, 13% in UC). Endoscopy was performed in 26.5% and 23.4% of CD and UC patients in the 12 weeks prior to biologic dose escalation, respectively [40]. A total of 233 (44.4%) patients discontinued a biologic therapy, with 79.4% having ≥1 means of objective disease assessment performed in the preceding 12 weeks and 42.5% having ≥2. CRP was the most utilised measure in CD (46.3%), whereas endoscopy was the most utilised for UC (39.7%). Endoscopy was performed in 33.1% of CD patients prior to discontinuation of a biologic therapy. Fcal was again poorly utilised (6.9% in CD, 8.2% UC). Cross-sectional imaging was significantly more common in CD than UC (MRI 14.4% vs. 0%, *p* < 0.001), with CT at 19.4% in CD vs. 8.2% in UC, *p* = 0.03). 

Adherence to three-monthly disease monitoring and its effect on outcomes was assessed in a real-world prospective multicentre study of 104 consecutive IBD patients commenced on adalimumab and followed-up for 12 months [39]. Adherence to clinical assessment across the 12 months was high, ranging from 81.3–87.7% in CD patients and 76.5–90.9% in UC patients. However, both CD and UC patients had roughly only 50% adherence with CRP measurements at 3 and 6 months and had progressive declines in adherence to 37.3% and 29.4% at 12 months, respectively. Adherence with FCAL measurement ranged from 22.7–31.3% in CD patients and from 17.6–56% in UC patients. A total of 21.5% of CD patients and 40.9% of UC patients underwent endoscopic assessment at 0–6 months, while 26.3% and 34.6% underwent endoscopic assessment at 6–12 months, respectively. 

The proportion of patients completing a similar regimen of a three-monthly review of clinical status and biomarkers was assessed in a retrospective observational study of 428 consecutive IBD patients commenced on adalimumab [41]. Clinical assessment at 3 months was 95.5% in CD patients and 94.3% in UC patients, while CRP and FCAL assessment was 70.6% and 25.4% and 64% and 33.3%, respectively. At 6 months, clinical assessment, CRP, and FCAL were 90.1%, 54%, and 24.6% in CD patients and 83.8%, 52.7%, and 13.5% in UC patients. Clinical assessment, CRP, and FCAL at 12 months were 95.6%, 55.2%, and 29% in CD patients and 88.5%, 51.9%, and 19.2% in UC patients, respectively. Clinical remission at 12 months was significantly higher in both CD and UC patients, demonstrating combined adherence at 3 months compared with non-adherent patients, with 63.6% vs. 43.3%, *p* = 0.001 for CD patients and 43.9% vs. 20.0%, *p* = 0.001 for UC patients. 

Further research is needed to elucidate the reason for sub-optimal adherence to interval disease assessments. While poor adherence is likely to be multifactorial, patient engagement and understanding of the need for tight disease monitoring is critical for the success of T2T approaches in the real world. This is likely to be particularly important among patients in clinical remission, for whom the importance of treating to a target of endoscopic remission will require ongoing engagement despite the absence of symptoms. 

**Table 3:** Proportion of patients completing objective disease assessments as part of tight disease monitoring.

Study, Year, Design	Population (No., UC/CD), Follow-Up	Interval of Objective Assessment	Proportion of Patients Completing Assessment at Relative Intervals	Outcome Associations
Al Khoury et al., 2021, retrospective observational [41]	428 pts, 338 (79%) CD, 90 (21%) UC	Three-monthly to 12 months	At 3 months follow-up Clinical: 95.5% CD, 94.3% UC CRP: 70.6% CD, 64% UC Fcal: 25.4% CD, 33.3% UC At 6 months follow-up Clinical: 90.1% CD, 83.8% UC CRP: 54% CD, 52.7% UC Fcal: 24.6% CD, 13.5% UC At 12 months follow-up: Clinical: 95.6% CD, 88.5% UC CRP: 55.2% CD, 51.9% UC Fcal: 29% CD, 19.2% UC	Clinical remission at 12 months associated with: Combined adherence at 3 months vs. non-adherence in CD, 63.6% vs. 43.3%, *p* = 0.001. Combined adherence at 3 months vs. non-adherence in UC, 43.9% vs. 20.0%, *p* = 0.001.
Wetwittayakhlang et al., 2022, prospective observational [39]	104 pts, 82 (79%) CD, 22 (21%) UC, consecutively recruited	Three-monthly to 12 months	At 3 months follow-up Clinical: 87.7% CD, 90.9% UC CRP: 54.9% CD, 50% UC Fcal: 23.5% CD, 18.2% UC At 6 months follow-up Clinical: 83.8% CD, 90% UC CRP: 46.3% CD, 50% UC Fcal: 31.3% CD, 25% UC At 12 months follow-up: Clinical: 81.3% CD, 76.5% UC CRP: 37.3% CD, 29.4% UC Fcal: 22.7% CD, 17.6% UC Endoscopy in first 6 months: 21.5% CD, 40.9% UC Endoscopy in second 6 months: 26.3% CD, 34.6% UC	Clinical remission at 12 months associated with: Early combined adherence vs. non-adherence in CD (70.2% vs. 29.8%, *p* = 0.007). Earlier dose optimisation of adalimumab associated with: Early combined adherence at 3 and 6 months in CD and UC (log-rank < 0.001).
Click et al., 2022, data from prospective longitudinal cohort [40]	525 pts, 375 (71.4%) CD, 150 (28.6%) UC	Within 12 weeks prior to dose escalation or cessation of biologic therapy	Prior to escalation of therapy (n = 292) ≥1 measure: 67.9% CD, 66.32% UC ≥2 measures: 33.5% CD, 42.9% UC CRP: 39.1% CD, 54.5% UC Fcal: 5.6% CD, 13% UC Endoscopy: 26.5% CD, 23.4% UC Prior to discontinuation of therapy (n = 233) ≥1 measure: 79.4% CD, 79.5% UC ≥2 measures: 44.4% CD, 38.4% UC CRP: 46.3% CD, 35.6% UC Fcal: 6.9% CD, 8.2% UC Endoscopy: 33.1% CD, 39.7% UC	N/A

CD, Crohn’s disease; CRP, C-reactive protein; Fcal, faecal calprotectin; No., number; Pts, patients; UC, ulcerative colitis.

### 4.5. Effect on Therapeutic Decision Making 

A further challenge of treating-to-target in IBD is how to incorporate the various treatment targets into therapeutic decisions. The limited number of treatment options and the potential for adverse effects mean decisions to alter IBD therapy require careful consideration [42,43]. Where MH is often not achieved, decisions to escalate treatment will need to be weighed against the relative paucity of therapeutic options available, particularly for patients in symptomatic remission who are otherwise clinically well. Randomised, controlled data are needed, demonstrating the long-term effects of MH before a treatment approach ultimately geared towards MH can be incorporated into real-world clinical practice [33]. Further, clear, evidence-based recommendations are needed for cases where MH is unable to be achieved despite adequately directed therapy. The identification of factors that predict the achievement of MH are likely to be useful for patient stratification and in the formulation of treatment algorithms [33]. In the interim, the decision to combine or escalate therapies in asymptomatic patients with endoscopic disease activity will remain complex and case-dependent.

Further research is also needed to guide the incorporation of therapeutic drug monitoring (TDM) into treat-to-target guidelines. Reactive TDM has been demonstrated to have a beneficial role in patients with a suspected loss of response to anti-TNF agents, leading to increased rates of both clinical and endoscopic remission [44,45]. Results from studies evaluating the benefits of proactive TDM, however, are mixed [46,47,48]. Further research is needed to elucidate the place of TDM in treat-to-target algorithms. 

### 4.6. Acceptability and Real-World Uptake

Patient and clinician acceptability of a treat-to-target approach is essential for its real-world uptake. An unselected cohort of 298 consecutive patients with IBD (144 CD, 136 UC, 18 unclassified IBD) in clinical remission within the Leeds Teaching Hospital NHS Trust catchment area were interviewed about a T2T approach [49]. A total of 66.2% rated a treat-to-target approach as acceptable, as defined by a Likert scale score ≥ 8. Avoidance of flare, hospitalisation, surgery, and colorectal cancer were rated as acceptable treatment aims. Participants with better adherence to current therapy were more likely to accept a treat-to-target approach (B = 0.16, *p* = 0.039) [49]. 

A south Australian study investigating the uptake of a treat-to-target approach in real-world practice found that of 246 patients with UC, only 85 (35%) were in combined clinical and endoscopic remission at the time of review [50]. In the remaining 65% of patients, clinician-related factors were the most frequently identified issue limiting the attainment of composite remission, specifically the failure to evaluate patients endoscopically. Of these patients, failure to seek and confirm endoscopic remission in patients in clinical remission was documented in 32% of cases, failure to assess endoscopic response to escalation of therapy in clinically active disease was seen in 20% of cases, and failure to endoscopically assess clinically active disease where there was no escalation of therapy was seen in 12% of cases. Comparatively, appointment or medication non-compliance was documented in only 17% of patients not in composite remission. Endoscopy was performed within 3 months in 47% of cases where clinically active disease was reported. Where clinically active disease was documented, a significantly higher proportion of patients underwent an assessment of disease activity with CRP rather than with endoscopy, i.e., 75% vs. 47%, *p* < 0.001 [50]. The real-world uptake of treating-to-target is likely to need increased acceptability on both patients’ and clinicians’ behalf. 

### 4.7. The need to Specifically Address Psychological Co-Morbidity in IBD 

Patients with IBD are disproportionately affected by psychological co-morbidity, especially in the form of anxiety and depressive disorders [51,52]. Up to a third of patients are affected by symptoms of anxiety and a quarter by symptoms of depression [53]. In a systematic review of 171 studies including more than 158,000 patients with IBD, the pooled prevalence of diagnosed anxiety and depression was 20.5% and 15%, respectively [54]. Periods of active disease are associated with a worsening of psychological symptoms; however, significant levels of anxiety and depression can exist even during periods of disease remission [55,56].

The presence of co-morbid anxiety and/or depression in IBD is negatively associated with QoL, as measured by multiple tools, including the IBD questionnaire (IBDQ) and short IBDQ (SIBDQ) [57]. The presence of these psychological co-morbidities is also associated with increased rates of both all-cause hospitalisation and IBD-related hospitalisation, as well as an increased rate of early re-admission post IBD-related hospitalisation [57,58,59]. Due to their significant impacts, these psychological co-morbidities require specific screening and management attention, in addition to the physical symptoms of IBD [57,60]. 

Psychological therapies including mindfulness and cognitive behaviour therapy (CBT) have been shown to reduce anxiety and depression and significantly improve quality of life for IBD patients [52,61,62]. Albeit in a limited number of studies, psychological therapies have also shown promise in improving medication adherence in IBD, a significant barrier to the implementation of successful T2T strategies [63,64]. Further research into IBD-tailored psychological therapy and the role of adjunctive psychotropic medications is needed to optimise the management of psychological comorbidity in patients with IBD [65].

## 5. Solutions to the Challenges 

### 5.1. Non-Invasive Objective Monitoring of Disease Activity—Biomarkers

The use of biomarkers in a T2T approach may help to stratify the need for endoscopic disease assessment and thus reduce the overall reliance on endoscopy. Reflecting this, the updated STRIDE-II guidelines include biomarkers as an intermediate target [36]. 

Using biomarkers to guide treatment intensity in a tight control approach can also lead to improved endoscopic and clinical outcomes. The was demonstrated in the CALM trial, where a timely escalation of therapy based on FCAL and CRP in addition to symptoms resulted in superior clinical and endoscopic outcomes than adjustments based on symptoms alone [66]. Two hundred and forty-four patients with active endoscopic Crohn’s disease were enrolled, and biomarkers were measured at weeks 11, 23, and 35. A total of 46% of patients managed in a tight control capacity achieved mucosal healing at 48 weeks, as defined by CDEIS < 4 with an absence of deep ulcers at 48 weeks, compared to 30% of patients in the standard clinical management group (adjusted risk difference 16.1 (95% CI 3.9–28.3; *p* = 0.01)). Further, tight control was associated with a significantly lower rate of CD-related hospitalisations compared with the standard management group (13.2 events/100 patient-years vs. 28.0, *p* = 0.021). 

A recent post hoc analysis of data from the VARSITY trial investigated the prognostic capacity of post-induction Fcal and CRP to predict clinical and endoscopic outcomes in patients with UC treated with a biologic therapy [14]. Biomarkers were taken at week 14 post induction with vedolizumab or adalimumab and compared with outcomes at 52 weeks, with results adjusted for the treatment arm (vedolizumab vs. adalimumab) and prior anti-TNF exposure. Patients with a post-induction FCAL of ≤250 μg/g were significantly more likely to achieve clinical remission at week 52 compared to patients with FCAL >250 μg/g (OR 4.03, 95% CI 2.78–5.85; *p* < 0.0001), and they were also significantly more likely to achieve endoscopic remission at week 52 (OR 4.26, 95% CI 2.83–6.40; *p* < 0.0001). Compared to a post-induction CRP of >5 mg/dL, a CRP of ≤5 mg/dL was significantly associated with clinical and endoscopic remission at 52 weeks, with OR 3.46, 95% CI 2.22–5.38; *p* < 0.0001 and OR 2.02, 95% CI 1.30–3.16; *p* = 0.0019, respectively. 

Issues with adherence to biomarker monitoring is noted in several studies, particularly with faecal biomarkers [39,40,41]. However, the stability of Fcal makes initiatives such as home-based sample collection feasible, with evidence of significant improvements in adherence and good correlation to reference collections being observed with these initiatives [67,68].

### 5.2. Non-Invasive Objective Monitoring of Disease Activity—IUS

Cross-sectional imaging offers potential solutions to the need for repeated disease assessment in a T2T algorithm. Magnetic resonance enterography (MRE) offers highly detailed cross-sectional imaging and many advantages in disease assessment; however, its expense precludes its repeated use in real-world clinical practice [69]. Provided adequate training and equipment are available, intestinal ultrasound (IUS) provides a radiation-free and non-invasive means of disease assessment that can be readily repeated in a tight monitoring capacity [70].

The advantages of IUS compared to endoscopy include a lack of bowel preparation and anaesthesia as well as the ability to assess for extra-luminal disease activity and transmural healing (TH) [71]. It is also comparatively cheap per instance of use. Akin to histological healing in UC, transmural healing in CD is not included as a formal target in the STRIDE guidelines based on a current lack of evidence of benefits [4]. The ability to measure TH remains a strength of IUS however, in anticipation of TH becoming an increasingly useful and prognostic target in CD. 

The accuracy of IUS in assessing CD was demonstrated in a prospective observational study of 60 consecutively enrolled patients with ileocolonic CD [72]. Enrolled patients underwent MRE, colonoscopy, and IUS within 1 week, and the sensitivity, specificity, and accuracy of IUS in evaluating disease features when compared with colonoscopy + MRE was assessed. IUS had a sensitivity of 92%, specificity of 100%, and accuracy of 96% for the presence of active disease (ulcers on colonoscopy), a sensitivity, specificity, and accuracy of 88%, 96% and 91%, respectively, for disease localisation, a sensitivity, specificity, and accuracy of 100%, 98%, and 98%, respectively, for the presence of fistulas, and a sensitivity, specificity, and accuracy of 100%, 96%, and 96%, respectively, for abscesses [72].

The correlation between IUS parameters and endoscopic disease assessment has been reviewed by multiple systematic reviews. Correlation is variable and disease-dependent, and the current evidence is limited by the use of non-established endoscopic indices in multiple studies [71,73]. Further research is needed to validate IUS parameters in predicting therapeutic outcomes and endoscopic disease activity and to standardise the terminology and disease parameters. 

### 5.3. Individualisation of Treatments 

The individualisation of treatment plans in accordance with patient-centred care is critical when considering a T2T approach. Treating to the target of endoscopic healing is unlikely to be universally applicable, whether due to feasibility or patient preference. 

The increased incorporation of patient-reported outcomes (PROs) in treatment targets is likely to help facilitate the uptake of treating-to-target in real-world clinical practice. PROs are psychometric instruments completed by patients to quantify symptoms and disease impact without the need for an interpretation of patient response by a clinician [74]. The careful development of PROs with thorough psychometric evaluation should be the target of future research with the aim of reliable and practical use in clinical practice. The incorporation of PROs in treatment targets is likely to significantly improve patient engagement, adherence, and the overall success of treating-to-target in real-world clinical practice. 

Owing to an aging population and an increasing incidence of IBD, the burden of IBD is increasing in the elderly population [75]. A T2T approach in the geriatric subgroup of patients with IBD is likely to be further complicated by additional treatment considerations and the need for the individualisation of approaches. The current reliance on endoscopy and the potential need for intensive treatments may preclude this from individuals on safety grounds [25]. Treatment can be further complicated by difficulties in applying data from trials of IBD therapies due to an under-representation of elderly patients, particularly in RCT’s [76]. This is likely to have an impact on the ability to generalise data on both the efficacy as well as the safety of newer-age medications such as biologics [77]. Further, issues such as polypharmacy, changes in pharmacokinetics, and co-morbidities in the elderly are likely to increase the propensity for adverse effects of advanced IBD therapies and the likelihood of complications of management [25]. Targets other than MH may be more appropriate in the elderly. Aiming for clinical remission and adequate QoL may well be the most acceptable targets in light of a reduced burden of investigations and potential treatment-related adverse effects. 

## 6. Conclusions and Future Needs

A T2T approach that is aimed at the achievement of endoscopically defined MH is likely to improve outcomes in patients with IBD. Numerous drawbacks to treating-to-target in IBD remain, however, that are likely to limit its current applicability to real-world practice. 

A validated definition of MH which optimally predicts clinically significant benefits in IBD currently remains unknown. Large, prospective trials demonstrating the safety and long-term benefits of MH are needed before treating-to-target can be routinely implemented and used to justify treatment decisions. Until a standardised and validated definition of MH exists, the real-world implementation of treating-to-target in IBD will remain limited. 

The incorporation of tight control into treatment decisions has been shown to improve outcomes. However, the assessment of the real-world feasibility of close monitoring is minimal and demonstrates suboptimal patient adherence. Patient acceptability of a T2T strategy appears to reflect the level of current disease control, though an increased acceptability of T2T is likely to be needed on both patients’ and clinicians’ behalf for T2T to be successful in real-world practice. 

Further research is needed to support the role of biomarkers and IUS in the timely assessment of disease activity in IBD. The identification of biomarker thresholds to guide more invasive disease assessment and/or treatment decisions is a feasible way of reducing the burden of endoscopy and increasing the applicability of T2T outside of IBD centres. Further standardisation of IUS parameters and the validation of IUS in predicting outcomes is similarly likely to ease the burden of endoscopy. Further data are needed in the correlation of IUS with endoscopic findings, which is also likely to be aided by increased standardisation of endoscopic mucosal assessment in IBD. 

## Figures and Tables

**Table 1:** Studies reporting long-term benefits of endoscopically defined mucosal healing.

Study, Year, Design	Population	Definition of MH, Proportion of pts	Outcomes Assessed	Key Findings
Long-term disease control				
Shah et al., 2016, meta-analysis of 13 prospective studies [16]	2073 pts with active UC	Multiple definitions among included studies	Long-term CR, long-term defined as ≥52 weeks post treatment and ≥6 months post assessment of MH	pOR of 4.50 (95% CI, 2.12–9.52; *p* < 0.0001) for achieving long-term CR in patients achieving MH compared to those not
Shah et al., 2016, meta-analysis of 12 prospective studies [17]	673 pts with active CD	Multiple definitions among included studies	Long-term CR, long-term defined as ≥50 weeks from study outset	pOR of 2.80 (95% CI, 1.91–4.10; *p* < 0.00001) for achieving long-term CR in patients achieving MH vs. those not
Colombel et al., 2011, retrospective analysis of previous RCT’s [18]	728 UC pts with MES ≥ 2, treated with IFX or placebo	MES	CR at week 30, CR at week 54	Lower MES at week 8 was associated with increased likelihood of CR at week 30, 71% MES 0, 51% MES 1, 23% MES 2, 9.7% MES 3, *p* < 0.0001 and week 54, 73% MES 0, 47% MES 1, 24% MES 2, 10% MES 3, *p* < 0.0001 among IFX-treated patients
Ferrante et al., 2013 [19]	172 pts with CD treated with IFX, AZA, or both	MH: absence of ulcers Present in: 48% of patients at week 26 of treatment	CS-free CR at week 50	MH at week 26 was associated with CS-free CR at week 50 with 56% sensitivity, 65% specificity, PLR of 1.60, and NLR of 0.67.
Avoidance of surgery				
Shah et al., 2016, meta-analysis of 13 prospective studies [16]	2073 pts with active UC	Multiple definitions among included studies	Avoidance of colectomy at ≥52 weeks post treatment commencement and ≥6 months post finding of MH	pOR of 4.15 (95% CI, 2.53–6.81; *p* < 0.00001) for avoiding colectomy
Colombel et al., 2011, retrospective analysis of previous RCT’s [18]	728 UC pts with MES ≥ 2, treated with IFX or placebo	MES	Avoidance of colectomy at 54 weeks	Lower MES at week 8 among IFX-treated patients associated with increased likelihood of avoiding colectomy, 95% MES 0, 95% MES 1, 83% MES 2, 80% MES 3, *p* = 0.0004
Schnitzler et al., 2009, retrospective observational cohort study [20]	214 CD pts on long-term IFX treatment with endoscopy before and during IFX therapy	Complete MH: absence of ulceration in patients who had ulcerations at baseline—present in 83 pts (45.4%) Partial MH: clear endoscopic improvement but with ulceration present—present in 41 pts (22.4%)	Avoidance of MAS, defined as any gut resection, stricturoplasty, or faecal diversion surgery during follow-up period—median (IQR) follow-up 68.7 (39.8–94.8) months.	Reduced need for MAS in patients demonstrating complete or partial MH compared to those not, 14.1% vs. 38.4% of patients, *p* < 0.0001
Frøslie at al., 2007, retrospective observational cohort study [21]	495 pts with newly diagnosed UC (354) or CD (141) with endoscopic assessment at baseline, 1 and 5 years	Definition of MH not stated. Present in 178 (50%) of UC patients and 53 (38%) of CD patients at one year.	Avoidance of colectomy at 5 years	Presence of MH at 1 year follow-up associated with significantly reduced risk of colectomy at 5 years, RR 0.22 (95% CI, 0.06–0.79; *p* = 0.02)
Long-term mucosal healing				
Shah et al., 2016, meta-analysis of 13 prospective studies [16]	2073 pts with active UC	Multiple definitions among included studies	MH at ≥52 weeks post treatment commencement and ≥6 months post finding of MH	pOR of 8.40 (95% CI, 3.13–22.53; *p* < 0.00001) for achieving long-term MH in patients achieving MH vs. those not
Shah et al., 2016, meta-analysis of 12 prospective studies [17]	673 pts with active CD	Multiple definitions among included studies	Long-term CR. Long-term defined as ≥50 weeks from study outset	pOR of 14.30 (95% CI, 5.57–36.74; *p* < 0.00001) for long-term MH in patients achieving MH vs. those not
Colombel et al., 2011, retrospective analysis of previous RCT’s [18]	728 UC pts with MES ≥ 2, treated with IFX or placebo	MES	Sustained mucosal healing at both weeks 30 and 54	Lower MES at week 8 associated with increased rate of sustained MH at both weeks 30 and 54, 77% MES 0, 54% MES 1, 21% MES 2, 6.7% MES 3, *p* < 0.0001 among IFX-treated patients
Af Björkesten et al., 2013, prospective observational study [22]	42 pts with active CD treated with IFX or adalimumab	MH: SES-CD 0–2. MH present in 10 (24%) patients at 3 months post therapy commencement.	Presence of MH at 1 year	Patients with MH at 3 months more likely to demonstrate MH at 1 year than those without, 70% vs. 17%, *p* = 0.01

AZA, Azathioprine; CD, Crohn’s disease; CI, confidence interval; CR, clinical remission; CS, corticosteroid; IFX, Infliximab; MAS, major abdominal surgery; MES, Mayo endoscopy subscore; MH, mucosal healing; NLR, negative likelihood ratio; pOR, pooled odds ratio; PLR, positive likelihood ratio; RCT, randomised, controlled trial; RR, risk ratio; UC, ulcerative colitis.

**Table 2:** Studies investigating the rate of achieving mucosal healing with use of serial endoscopy.

Study, Year, Design	Population (No., UC/CD), Follow-Up	Presence of Baseline Endoscopic Activity (No., %)	No. of Endoscopic Assessments, No. (%) pts Undergoing	No. of Therapy Adjustments Made	Definition of MH, Definition of ER, No. (%) Achievement	Associations
Bouguen et al., 2014, retrospective observational study [34]	60 pts, 100% UC, median follow-up 76 weeks	45 (75%)	2: 26 (43%) 3: 26 (43%) 4: 8 (13%) Median interval between consecutive endoscopies 25 weeks (IQR, 16–42 weeks)	51 adjustments made within the 45 pts with endoscopic disease activity	MH: MES = 0. MH: 27 (60%) pts with baseline endoscopic disease activity	MH associated with: Post-endoscopy adjustments in medical therapy made in the case of persistent endoscopic activity (HR 9.8, 95% CI 3.6–34.5; *p* < 0.0001).
Bouguen et al., 2014, retrospective observational study [35]	67 pts, 100% CD, median follow-up 62 weeks	67 (100%)	2: 40 (60%) 3: 21 (31%) 4: 6 (9%) Median interval between consecutive endoscopies 24 weeks (IQR, 17–38 weeks)	72 adjustments made as a result of endoscopic findings of ulceration	MH: absence of any ulcers in GIT. ER: downgrading of deep ulcers to superficial ulcers or the disappearance of superficial ulcers. MH: 34 (50.7%) pts, ER: 41 (61.1%) pts	MH associated with: <26 weeks between endoscopic procedures (HR 2.35; *p*= 0.035), adjustment to medical therapy when MH was not observed (HR 4.28; *p* = 0.0003).
Meade et al., 2023, retrospective observational study [36]	50 pts, 100% CD	50 (100%)	2: 50 (100%) Interval between endoscopies not stated	0	MH: SES-CD ≤ 2 ER: >50% reduction in SES-CD MH: 25 (50%) ER: 35 (70%)	Treatment failure associated with: Failure to achieve MH (HR 11.62, 95% CI 3.33–40.56; *p* = 0.003), failure to achieve ER (HR 30.30, 95% CI 6.93–132.30; *p* < 0.0001).
Mao et al., 2017, retrospective observational study [37]	272 pts, 100% CD Median follow-up 33 months (IQR 27–38 months).	272 (100%)	2: 272 (100%) 3: 154 (56.6%) 4: 69 (25.3%) 5: 26 (9.6%) 6: 10 (3.6%) 7: 4 (1.5%) Median interval between consecutive endoscopy 24 weeks (IQR: 17–38 weeks).	237 adjustments made as a result of endoscopic findings of ulceration	MH: mucosal activity score of 0–2 MH: 126 (46.3%) endoscopic score system adopted from Af Björkesten et al. [38], mucosal activity scored from in most affected area.	MH associated with: <26 weeks between endoscopic procedures (HR 1.56; 95% CI 1.05–3.39; *p* = 0.03), adjustment of medical therapy when MH was not achieved (HR 2.07; 95% CI 1.26–2.33; *p* < 0.01), CRP normalisation within 12 weeks (HR 3.23; 95% CI 1.82–5.88; *p* < 0.01).
Wetwittayakhlang et al., 2022, prospective, observational study [39]	104 pts, 82 (79%) CD, 22 (21%) UC, consecutively recruited	70.6% CD 81.3% UC	2 (relative proportions of study population not stated)	Not stated	MH: not stated MH at 6 months: 46.2% of CD pts with baseline endoscopic activity. 25% of UC pts with baseline activity MH at 12 months: 44.4% of CD pts with baseline endoscopic activity. 33% of UC pts with baseline activity	Not stated

CD, Crohn’s disease; CI, confidence interval; ER, endoscopic response; GIT, gastrointestinal tract; HR, hazard ratio; IQR, interquartile range; MES, Mayo endoscopic sub score; MH, mucosal healing; No., number; Pts, patients; SES-CD, simple endoscopic score for Crohn’s disease; UC, ulcerative colitis.

## Data Availability

No new data were created or analysed in this study. Data sharing is not applicable to this article.
